# Combining Wearable Devices and Mobile Surveys to Study Child and Youth Development in Malawi: Implementation Study of a Multimodal Approach

**DOI:** 10.2196/23154

**Published:** 2021-03-05

**Authors:** Onicio Leal Neto, Simon Haenni, John Phuka, Laura Ozella, Daniela Paolotti, Ciro Cattuto, Daniel Robles, Guilherme Lichand

**Affiliations:** 1 Department of Economics University of Zurich Zurich Switzerland; 2 College of Medicine University of Malawi Lilongwe Malawi; 3 ISI Foundation Turin Italy; 4 Department of Computer Science University of Torino Turin Italy; 5 Department of Psychology University of Alberta Edmonton, AB Canada

**Keywords:** child development, wearables, participatory surveillance, proximity sensors, mobile surveying

## Abstract

**Background:**

Multimodal approaches have been shown to be a promising way to collect data on child development at high frequency, combining different data inputs (from phone surveys to signals from noninvasive biomarkers) to understand children’s health and development outcomes more integrally from multiple perspectives.

**Objective:**

The aim of this work was to describe an implementation study using a multimodal approach combining noninvasive biomarkers, social contact patterns, mobile surveying, and face-to-face interviews in order to validate technologies that help us better understand child development in poor countries at a high frequency.

**Methods:**

We carried out a mixed study based on a transversal descriptive analysis and a longitudinal prospective analysis in Malawi. In each village, children were sampled to participate in weekly sessions in which data signals were collected through wearable devices (electrocardiography [ECG] hand pads and electroencephalography [EEG] headbands). Additionally, wearable proximity sensors to elicit the social network were deployed among children and their caregivers. Mobile surveys using interactive voice response calls were also used as an additional layer of data collection. An end-line face-to-face survey was conducted at the end of the study.

**Results:**

During the implementation, 82 EEG/ECG data entry points were collected across four villages. The sampled children for EEG/ECG were 0 to 5 years old. EEG/ECG data were collected once a week. In every session, children wore the EEG headband for 5 minutes and the ECG hand pad for 3 minutes. In total, 3531 calls were sent over 5 weeks, with 2291 participants picking up the calls and 984 of those answering the consent question. In total, 585 people completed the surveys over the course of 5 weeks.

**Conclusions:**

This study achieved its objective of demonstrating the feasibility of generating data through the unprecedented use of a multimodal approach for tracking child development in Malawi, which is one of the poorest countries in the world. Above and beyond its multiple dimensions, the dynamics of child development are complex. It is the case not only that no data stream in isolation can accurately characterize it, but also that even if combined, infrequent data might miss critical inflection points and interactions between different conditions and behaviors. In turn, combining different modes at a sufficiently high frequency allows researchers to make progress by considering contact patterns, reported symptoms and behaviors, and critical biomarkers all at once. This application showcases that even in developing countries facing multiple constraints, complementary technologies can leverage and accelerate the digitalization of health, bringing benefits to populations that lack new tools for understanding child well-being and development.

## Introduction

### Background

Research on many determinants of child development, including its underlying biological mechanisms, has accelerated at a fast pace over the last decade. It is now well known that early childhood development is a maturational and interactive process, resulting in an ordered progression of perceptual motor, cognitive, language, socioemotional, and self-regulation skills [1,2]. It is also clear that early childhood nutrition crucially affects educational outcomes [3] that are, in turn, important for adult productivity and wealth. Having said that, while a lot is known about potential critical or sensitive periods in child development, we know much less about how the effects of different interventions interact, either within or across critical periods [3-5]. For instance, we know that decreasing malnutrition from ages 2 to 4 years leads to a higher likelihood of future employment [6]. We also know that early stimulation from ages 0 to 3 years leads to a higher likelihood of future employment [7]. However, the following question is important: do the two interventions combined have effects that are larger than the sum of its parts, because of complementarities, or are they substitutes, partly crowding out the effects of each other?

In addition, knowledge on the impacts of disease outbreaks on child development is still scarce, particularly when it comes to infection susceptibility disrupting the environment in which children grow [8]. Specifically, on the impact of infectious diseases, close proximity between caregivers and children under 5 years of age could increase the exposure risk in case caregivers fall ill [9]. Moreover, the consequences of disease outbreaks might interact with other features of child development, such as nutritional status, since disease burden can affect nutrition requirements to sustain child development under adverse conditions [10,11].

Panel studies in developing countries typically employ face-to-face data collection (ie, traditional survey instruments), which happens at a low frequency [12,13]. The Demographic and Health Survey, for instance, is updated only every 3 to 5 years. However, studying phenomena, such as those cited above, requires reliable high-frequency data. To accommodate this need, panel designs need to be reimagined to create better data streams.

Today’s technologies allow collecting a multiplicity of users’ features at scale and at a higher frequency and lower cost than face-to-face data collection, even if typically restrained to developed country settings. If their application could be extended to poor countries to track child development throughout different stages of their lives, such data could give us more power to evaluate interventions and improve causal inferences, and could help identify underlying mechanisms [14]. In particular, having access to high-frequency data on children’s biomarkers could allow for timely responses in public health (such as early warning systems), with immediate potential to decrease child mortality and morbidity, and to increase children’s cognitive development [15]. For instance, electroencephalography (EEG) is considered an optimal neuroimaging technique to record developmental changes during childhood [16]. Such markers of cognitive development include changes in oscillatory rhythm frequencies responsible for brain connectivity and cognitive functions [17] Moreover, having access to such data would allow learning about interactions between critical periods, as in the examples of nutrition and early stimulation mentioned previously. Importantly, if the frequency of data is high enough, it has the potential to feed machine learning models that predict child-level impacts of different interventions, allowing policymakers to customize child development programs at a high frequency.

While some of those technologies, including wearables, have been applied to track children’s conditions in developing country settings, this has typically been done either (1) in isolation, rather than combining multiple data streams, or (2) at a low frequency. The problem of the former is that each data stream in isolation has important limitations, for example, symptoms might be underreported in phone surveys, wearable readings might correspond to a multiplicity of states when considered in isolation, and contact patterns might be insufficient to characterize disease transmission dynamics. The problem of the latter is that above and beyond being multidimensional, the dynamics of child development are complex. It is the case not only that no data stream in isolation can accurately characterize it, but also that even if combined, infrequent data might miss critical inflection points and interactions between different conditions and behaviors.

Instead, multimodal approaches have been shown to be a promising alternative for high-frequency monitoring, combining different inputs of noninvasive biomarkers and creating multiple angles to understand health and clinical outcomes [18,19]. These data signals also include other types of data streams that can help understand more integrally the different aspects in each patient or subject. Despite the potential, there is a lack of studies showing the feasibility of high-frequency data collection in poor settings, which combines a multimodal approach to track child development at a high frequency.

### Aim

The aim of this study was to describe a multimodal approach combining noninvasive biomarkers, social contact patterns, mobile surveying, and face-to-face interviews in order to validate technologies that help us better understand child development, as well as epidemiological events at the community level.

## Methods

### Study Design, Location, and Timeline

We carried out a mixed study based on a transversal descriptive analysis and a longitudinal prospective analysis in Malawi. The field work took place in the following four villages in Dowa District: Mdoliro, Chidothi, Mtalanje, and Mkuwani. A pilot was run from October 1 to October 31, 2019. In this pilot, the team sampled villagers and users as described in Table 1. From November 1 to November 8, 2019, the field work team prepared the activities on the ground. Training sessions were conducted with health surveillance assistants and community watchers from selected villages. In this period, local authorities and stakeholders, such as the District Health Office, the District Health Management Team, and the District Emergency Committee, were involved. Moreover, a demonstration for village chiefs was performed. From November 22 to December 23, 2019, mobile surveys using interactive voice response (IVR) calls and text messages were started. In parallel, the internal team of researchers and surveyors ran fieldwork activities from November 29 to January 10, 2020. A research assistant supervised this fieldwork, making weekly spot checks for over a month. Wearable sensors were used from December 6, 2019, to January 10, 2020. The end-line face-to-face survey was conducted from January 6 to January 12, 2020.

**Table 1:** Sampling for the implementation phase.

Mode of data collection	Wearable devices (children 0-5 years old), n	Wearable proximity sensors (children + teenagers + adults), n	Face-to-face interviews (caregivers), n
Village 1	27	99	61
Village 2	12	N/A^a^	58
Village 3	21	N/A	61
Village 4	22	N/A	55
Total	82	99	235

^a^N/A: not applicable.

### Sampling

In each village, children were sampled to use wearable devices. Additionally, wearable proximity sensors to elicit the social network were deployed in one village. Table 1 provides details about the sampling strategy.

### Ethical Aspects

A key ethical concern is the protection of human subjects from physical or psychological harm. All participants in the study were fully informed about the nature and purpose of the research and their requested involvement. Only participants who provided written consent (documented) were included in the research. In the case of children (subjects under 10 years old), consent was obtained from their guardians. In the case of adolescents (subjects between 10 and 19 years old), consent was obtained from both themselves and their guardians. Specific safeguards were in place to protect the safety (both physical and psychological) of respondents and those collecting the data. Personal identification information, including the names of the children, the names of the caregivers, and the phone numbers of the caregivers, were only accessible to local field workers and to one research associate in Malawi. No researcher was able to see any personal identification information as data sets were anonymized. Before the study, every participant was informed about the content of the study, including the objective of the study and the different types of data collected. Thereafter, every participant read a consent form (in both Chichewa and English) and signed it to indicate that they agreed to participate or let their children participate in this study. For those who could not read and write, our field workers read the content of the consent form to the participant and the participant provided his/her fingerprint to indicate consent. Enumerators were trained in collecting sensitive information, including information about children and health. Data collection tools, including wearable devices, were tested and designed in a way that was culturally appropriate (eg, no stigma) and did not create distress for respondents (eg, not invasive). Data collection visits were organized at the appropriate time (eg, did not disturb the regular life of study participants) and place to minimize the risk to respondents. Enumerators were able to provide information on how individuals in situations of risk can seek support. Respondents were always given the option to withdraw from the study at any time. All data from the study were kept without identifiers to protect subject privacy. No data generated by this study, even if deidentified, can be exploited for commercial purposes by any of the counterparts involved. For the inception phase, two institutional review board (IRB) approvals were obtained. One was obtained from the Ethics Committee at the University of Zurich (OEC IRB #2018-046), and the other was obtained from the College of Medicine Research and Ethics Committee at the University of Malawi (P.10/19/2825).

### Multimodal Approach

#### Wearables: Electrocardiography Pads

**Figure 1:**
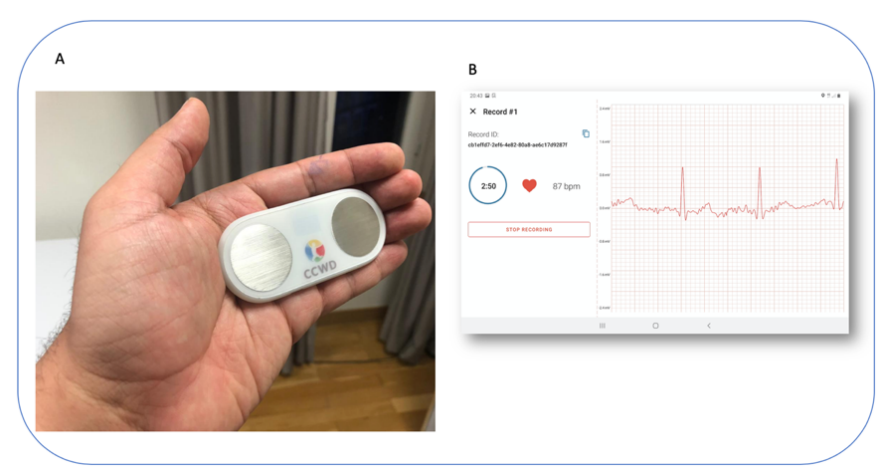
Electrocardiography pads with electrodes (A) and the tablet interface collecting the data (B).

Electrocardiography (ECG) pads (Figure 1) collect ECG data at a 500-Hz sample rate in a resting state. ECG is a recording of the electrical activity of the heart using electrodes placed on the skin. The signal is described in a graph of voltage versus time. The electrodes detect the small electrical changes that are a consequence of cardiac muscles followed by repolarization during each cardiac cycle (eg, heartbeat). The reason for including ECG data is to understand through heart rate and heart rate variability if identified patterns could be related to infectious diseases, as an increase in heart rate is considered a proxy when the body temperature increases.

During data collection, the study participants held the pad with their hands in a resting state and placed their thumbs on the metal electrodes for around 3 minutes. For small children who could not hold the pad, the health surveillance assistants and volunteers put two clips with electrodes on the child’s wrists, connected the clips to the pad with a cable, and waited for 3 minutes. The ECG data appeared on an app on a tablet, and the data were stored on the tablet, which could be later uploaded to the cloud.

#### Wearables: EEG Headbands

**Figure 2:**
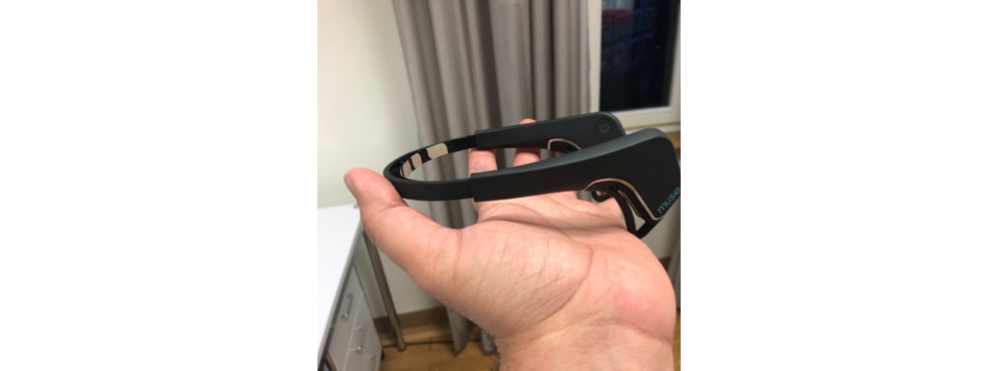
Muse headband used to collect electroencephalography data from children.

Muse headbands (Figure 2) were used to collect EEG data (research resource identifier: SCR_014418; provided by Interaxon). It is a portable scalp EEG system that can be used to measure brain activity. It is battery powered and has four active electrodes located at 10 to 20 coordinates (TP9, AF7, AF8, and TP10), a common mode reference, and a driven right leg. Muse headsets initially oversample EEG data and subsequently down sample the data to yield a selectable output at a sampling rate of 220 Hz to 500 Hz, with 2 µV (root mean square) noise. The input range of the AC-coupled signal (low cutoff at 1 Hz) is 2 mV [20-22].

The main research question that we were interested in is whether EEG data can show proxies that could be related to cerebral malaria, which has a high incidence in the country or whether those metrics could be related to cognitive development, enabling systematic long-term monitoring in children at the community level.

During recording sessions, health surveillance assistants and volunteers placed the headband on the child’s head, fitting the frontal electrodes above the eyebrow bridge and adjusting the posterior electrodes behind each mastoid. A block of 5 minutes of EEG data was recorded for each child while in a resting state with eyes open. The size of the headband could be adjusted to fit the size of the child’s head. For very young children whose head circumferences were too small to fit the headband, an elastic gauze material was used to wrap up around the headband to maintain firm connectivity. The EEG data were shown on an app on a tablet, and the data were stored on the tablet and later synced to an encrypted server for retrieval and analysis.

Both EEG and ECG signals were integrated in a mobile app used on a tablet by health surveillance assistants and community watchers. Additionally, portable solar power kits were distributed to make sure the devices would be recharged when necessary without relying on local electricity availability.

#### Wearables: Proximity Sensors

**Figure 3:**
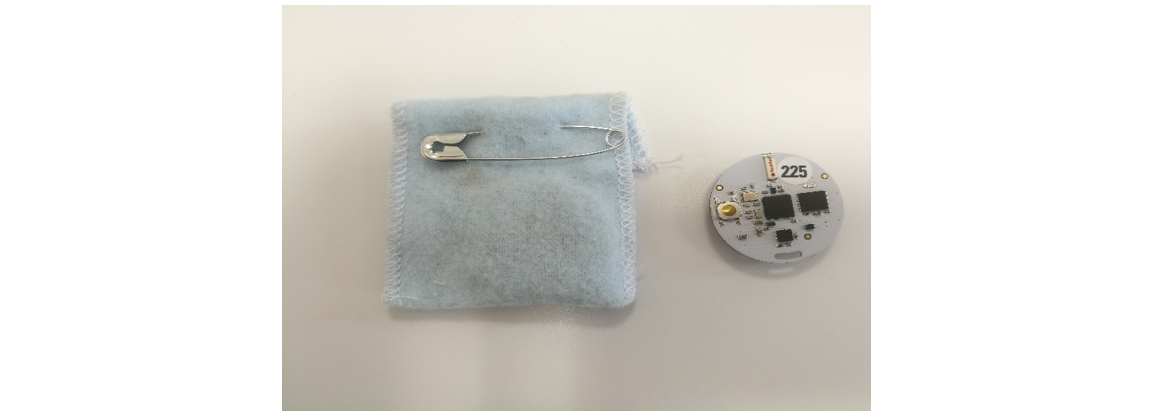
Proximity sensors and fabric pockets used to track social contact patterns.

Proximity sensors (Figure 3) using low-powered radio frequencies have been used in different settings, such as hospitals [23], households [24], and schools [25]. In Africa, proximity sensors have been applied in studies to characterize contact networks in households [26], and more recently, they have been used in households and schools [24,25]. Sensors provide contact data with high temporal resolution that can be used to investigate plausible characteristics of infection spread on network structures weighted by frequency and duration of contact [25,27]. Sensors exchange ultralow-power radio packets in a peer-to-peer fashion [28]. We considered that “contact” occurred between two individuals during a time slice duration of 20 seconds in a range of 1 to 1.5 m if the proximity sensors exchanged at least one radio packet during that interval. After contact is established, it is considered ongoing as long as the devices continue to exchange at least one packet for every subsequent 20-second interval [28]. All participants wore the device pinned to the front of a blouse/shirt in the chest area, and only face-to-face proximity relations were detected as the human body acts as a radiofrequency shield at the carrier frequency used for communication. Each device has a unique identification number that was used to link the information on the contacts established by the person carrying the device with his/her profile.

As a research aspect, we were interested in social network structures at the community level to help understand when and how a disease would spread through the villages. Additionally, we wanted to assess if it is possible to rely on network sensing to evaluate the effect of nudges sent during the study.

#### Mobile Surveying

A mobile-based system of data collection, where individuals were asked to participate in a survey via IVR, was used as an additional data collection layer [29]. Respondents were asked to report their personal assessments of the condition of their children. There were the following five different survey modules over the course of 5 weeks: (1) medical symptoms, (2) nutrition, (3) wash and habits, (4) early stimulation, and (5) child development. For symptoms, a syndromic protocol was used [30,31], where the occurrences of the following clinical outcomes were collected: fever, headache, diarrhea, rash, joint pain, sore throat, cough, body aches, itching, shortness of breath, and red eyes. For nutrition, the system collected information on food intake, vegetable and fruit intake, protein-rich food intake, food stocks, and breastfeeding. For wash, the reported information involved water availability and quality, available toilet facilities, and hand washing with soap. For early stimulation, we collected information on books and toys that might be available for a child, times when the child is left alone or in the care of another child, and activities that parents perform with the child, such as singing songs to the child. Finally, for child development, we collected information on the different types of activities that the child is currently able to perform. The survey questions and modules differed for children at different ages [32-34].

During the 1-month implementation, the selected participants received a call on their mobile phones every Friday for 5 weeks. One day before the phone call, they received an SMS text message that reminded them of the upcoming call [35-37]. If they picked up the call, they answered the survey questions by pressing different buttons on their phones. If they missed the call, they received another call later. They could call back the number to complete the survey at any time without any charge.

Besides mobile surveying by IVR, we sent nudges by SMS text message to the participants. By sending nudges on different topics, we wanted to test how participants react to the messages and whether there is any change in their behavior in response to the provided information (ie, treatment effect of nudges). It was decided to randomly split each of the four villages into two equal groups (control and treatment). The individuals in the treatment group received the nudges every week for 4 weeks. Conversely, the individuals in the control group did not receive the nudges. The weekly nudges asked caregivers to take their children to the health or nutrition clinic for check-ups, requested households to talk with other households about handwashing behavior, or asked caregivers to take their children on play dates with children from other households.

#### Face-to-Face Surveys

This study included the administration of (1) a light census and (2) a follow-up survey. These two data collections facilitated the interpretation of data coming from the devices.

First, in order to figure out who is eligible for the study and sample participants, the team performed a census survey in the four selected villages before the inception phase started. The survey only collected data about demographic information of the households and information about the medical situation of the children in the past 4 weeks, as well as their functioning mobile phone number and their willingness to receive SMS text messages/IVR calls.

After the fieldwork activities, the team deployed another face-to-face survey to collect information about child development, including health, wash, nutrition, education, child protection, and environmental data. Similarly, this follow-up survey collected reported information on device usability and acceptability, and similar ethical concerns that the population might raise because of the deployment of technological devices. We were interested to understand how the population receives a mobile message and if individuals share this information within the household and with neighbors. For this, we measured the ratio of information received through phone messages during the face-to-face data collection among segments of the target population that were not sent phone messages.

The follow-up survey contained the following four sections: demographics of the households and measured children; questions about the Child Development Study, including the reactions of the participants toward the study and the ethical concerns of the study; questions about the user experiences of the different wearable devices; and questions about the medical symptoms and medical treatments of the targeted children.

### Data Analysis

Descriptive analyses were undertaken showcasing the adherence and number of sessions involving wearable devices. For EEG data, we performed a grand average frequency spectra analysis evaluating the frequency range distribution. For proximity sensor data, we created a social contact matrix to evaluate relevant contacts during the study period. In addition, the contact patterns were compared with nudges sent by the IVR through a time series. For the end-line survey, we performed linear regression for medical symptoms. In-depth analyses were not the objective in this study and will be covered in future publications.

## Results

### Participation Characteristics

During the implementation, 82 EEG/ECG data entry points were collected in the four villages (Table 2). The sampled children for EEG/ECG were 0 to 5 years old. EEG/ECG data were collected weekly, meaning that health surveillance assistants/volunteers used the wearable devices to collect data on the children once a week. For every collection session, the children needed to wear the EEG headband for 5 minutes and needed to wear the ECG hand pad for 3 minutes. We report the frequency at which EEG and ECG data were collected in Tables 2 and 3, respectively.

**Table 2:** Frequency table for electroencephalography data (weeks).

Number of successful sessions per child	Frequency	Percentage	Cumulative
0	5	6.10	6.10
1	23	28.05	34.15
2	11	13.41	47.56
3	18	21.95	69.51
4	25	30.49	100
Total	82	100	N/A^a^

^a^N/A: not applicable.

**Table 3:** Frequency table for electrocardiography data (weeks).

Number of successful sessions per child	Frequency	Percentage	Cumulative
0	2	2.44	2.44
1	22	26.83	29.27
2	10	12.20	41.46
3	21	25.61	67.07
4	27	32.93	100
Total	82	100	N/A^a^

^a^N/A: not applicable.

### Outcomes

**Figure 4:**
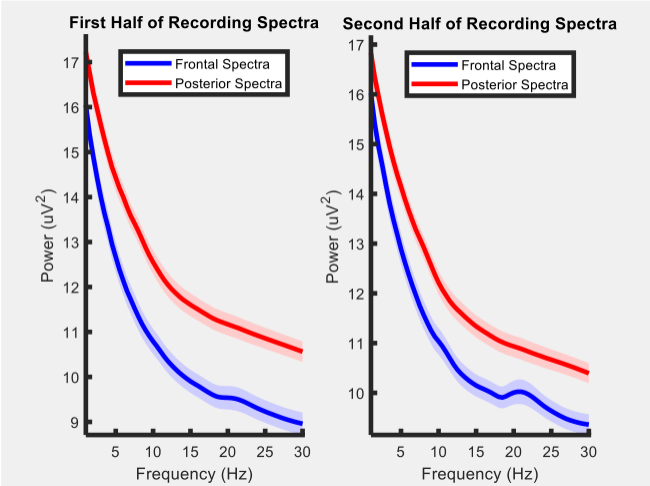
Grand average electroencephalography spectra for frontal and posterior electrodes for the first and second half of the recording period.

**Figure 5:**
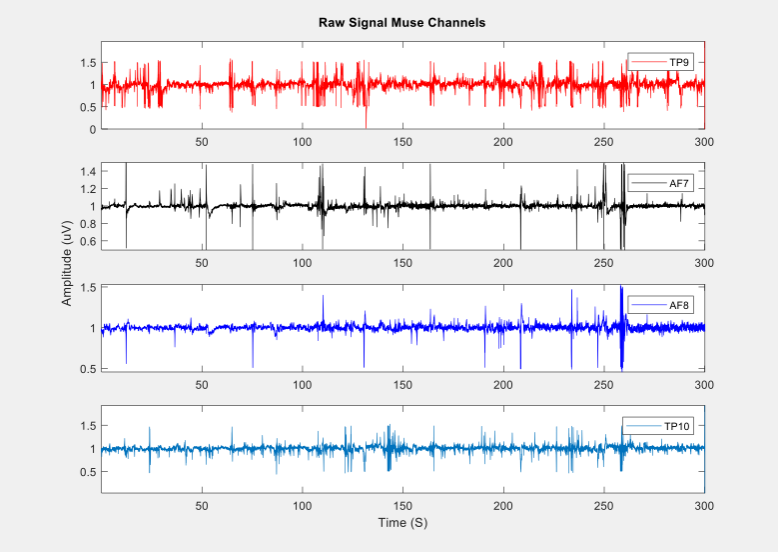
Sample of raw electroencephalography data at the frontal and posterior Muse electrodes.

**Figure 6:**
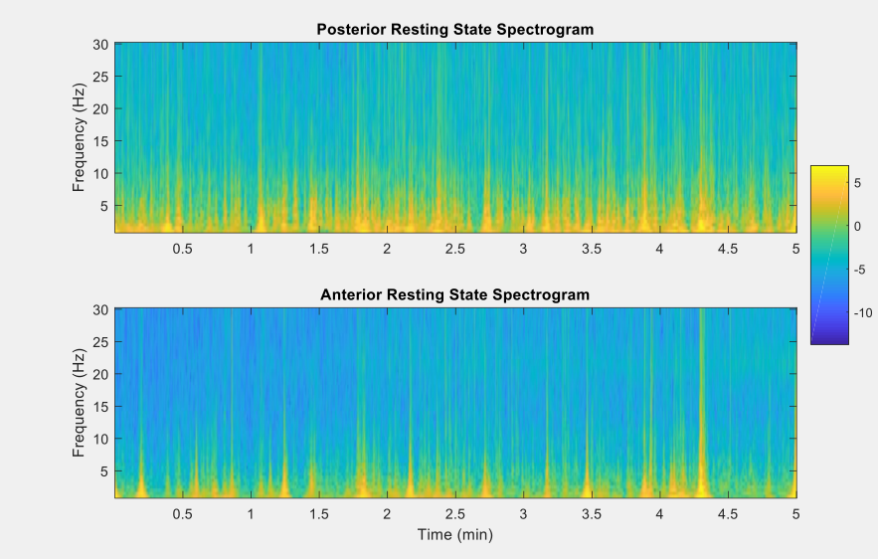
Electroencephalography spectrogram in the posterior and anterior regions across time.

Regarding EEG data collection, the goal of this study was to measure the feasibility of the Muse headband in recording the signal from the children. Figure 4 shows the grand average EEG spectra for the frontal and posterior Muse sensors, demonstrating that it is possible to collect reliable signals from our sample. Figure 5 presents a sample of raw EEG data for an entire recording period. The raw signal is displayed across frontal and posterior channels, showing changes in EEG over time and movement/blink artifacts. Figure 6 presents the EEG spectrogram for frontal and posterior electrodes, showing an overall trend of higher oscillatory activity in low frequencies over time. Taken together, these figures suggest that reliable EEG recordings can be obtained using this portable Muse methodology.

**Figure 7:**
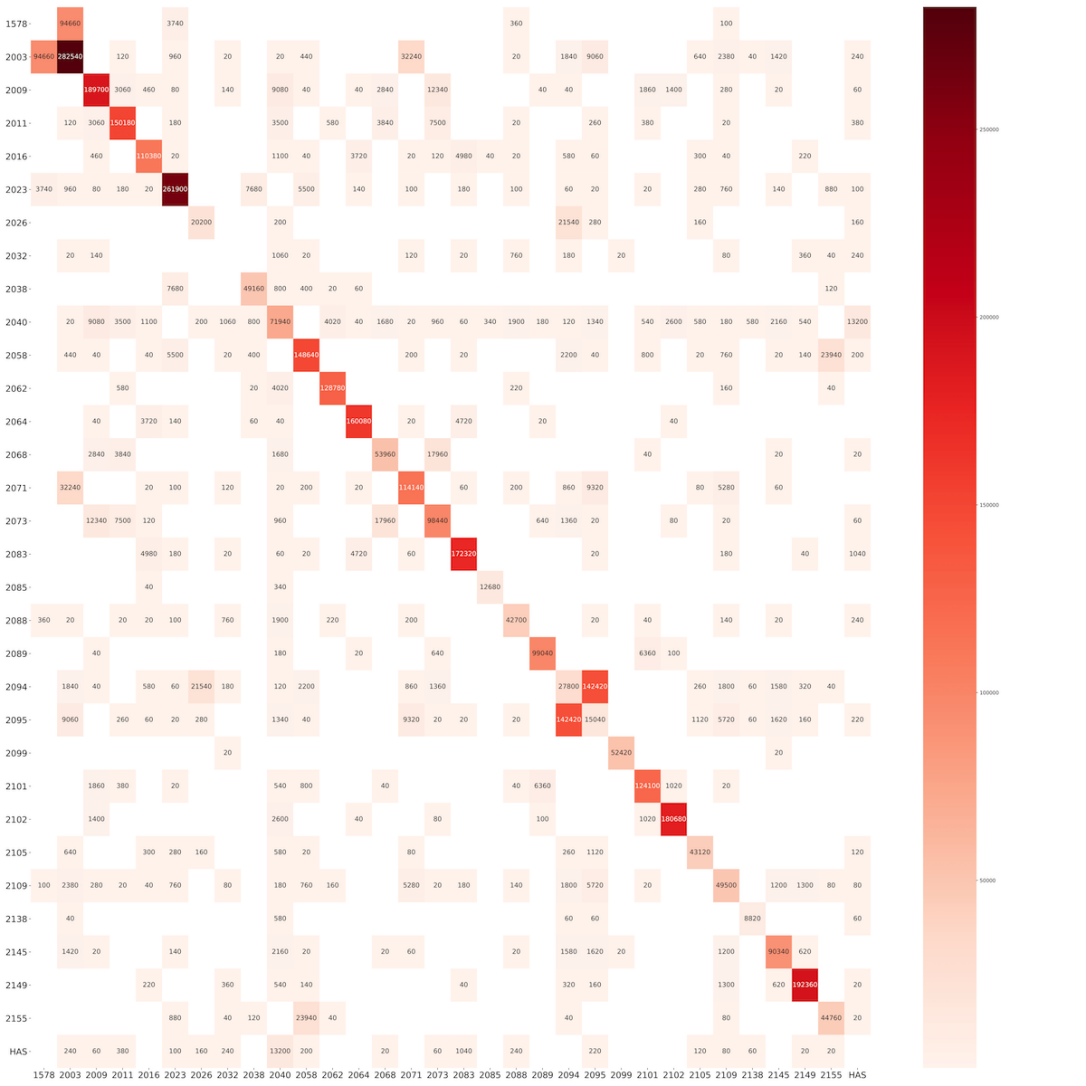
Intrahousehold and interhousehold total time in proximity (in seconds) registered by proximity sensors. The numbers on the Y axis and X axis represent the household ID. Each square represents the social contacts between the different households on the Y axis and the household on the X axis. The number in the square is the total time in proximity (in seconds). The numbers in the diagonal cells represent the total time in proximity among the members in the same household.

For the proximity sensors, the matrix (Figure 7) shows the intrahousehold and interhousehold social contacts.

In total, 3531 calls were sent over the 5 weeks the project was live, with 2291 people picking up the calls and 984 answering the consent question. In total, 585 people completed the surveys over the 5 weeks. This implies a pickup rate of 64.88% (2291/3531), a completion rate after pickup of 25.53% (585/2291), and a completion rate after answering the consent question of 59.5% (585/984). Completion rates dropped for each village after the first week, regardless of the incentive provided both in that week and the following weeks.

First, in order to understand if IVR surveys are feasible, we studied phone ownership in the reference area. Phone ownership ranges from 56% in Mkuwani to 81% in Mdoliro. The probability of owning a phone might be related to other characteristics of a household.

**Figure 8:**
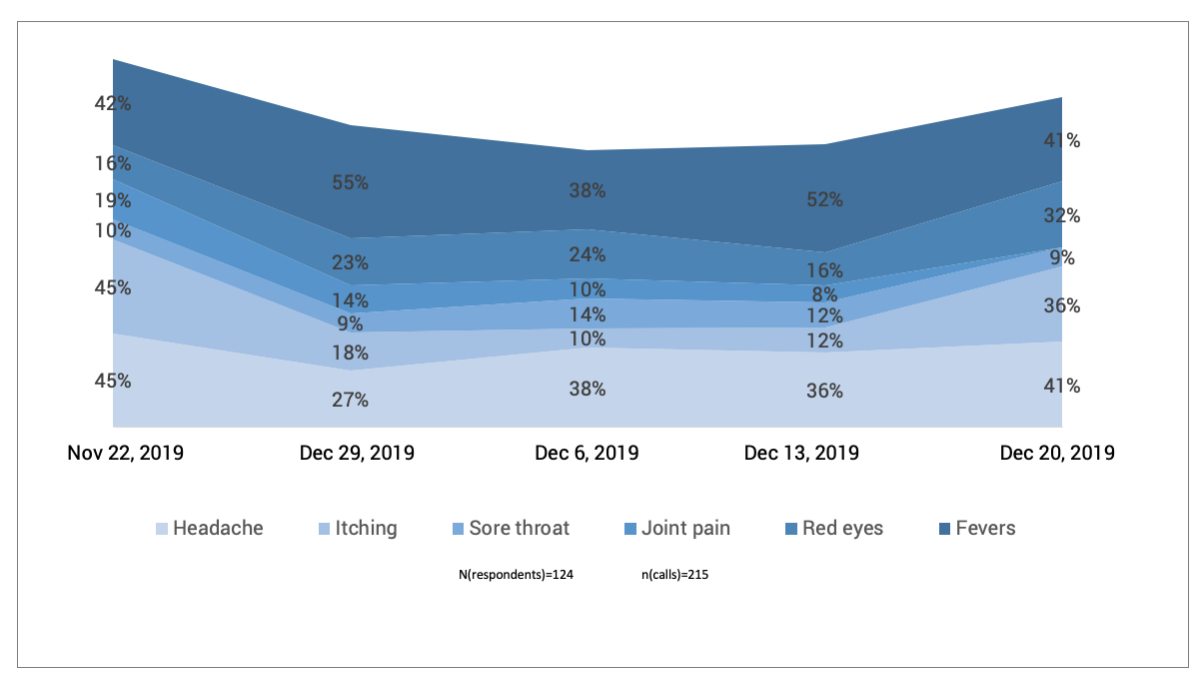
Temporal proportion distribution of most reported symptoms in the studied villages.

**Figure 9:**
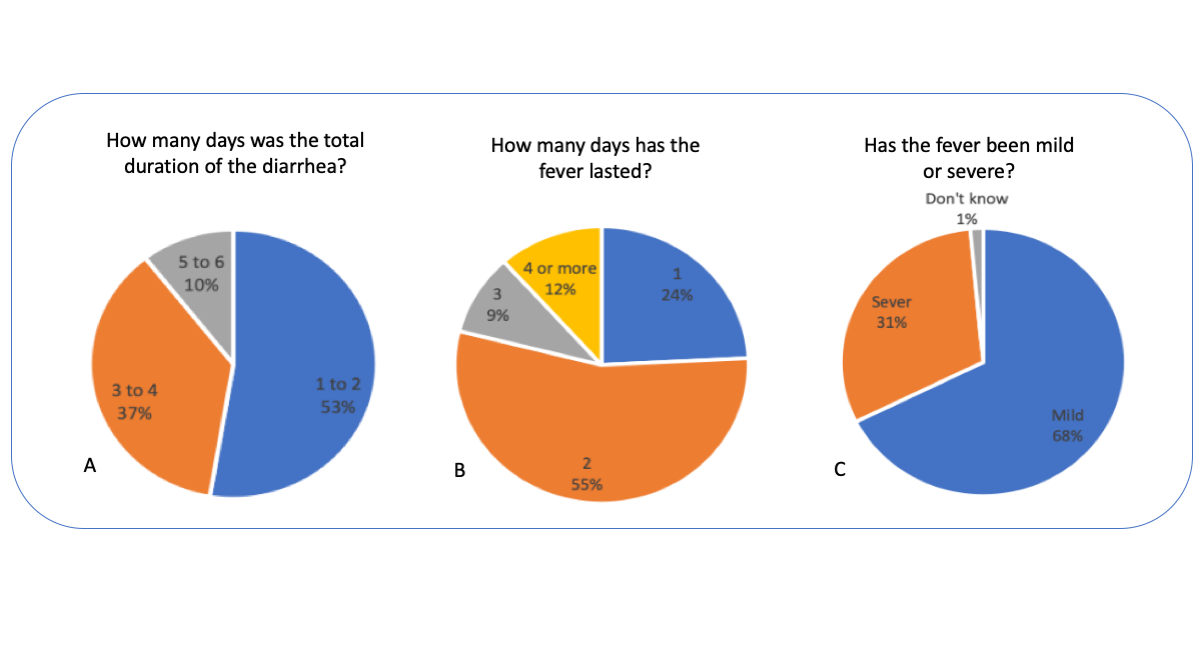
Severity of symptoms collected during mobile surveying.

Regarding the symptoms collected during the mobile surveying, Figure 8 shows the temporal proportion distribution along the study period. As shown in Figure 9, data on the severity of symptoms were collected, which can help distinguish between minor issues and major diseases.

**Figure 10:**
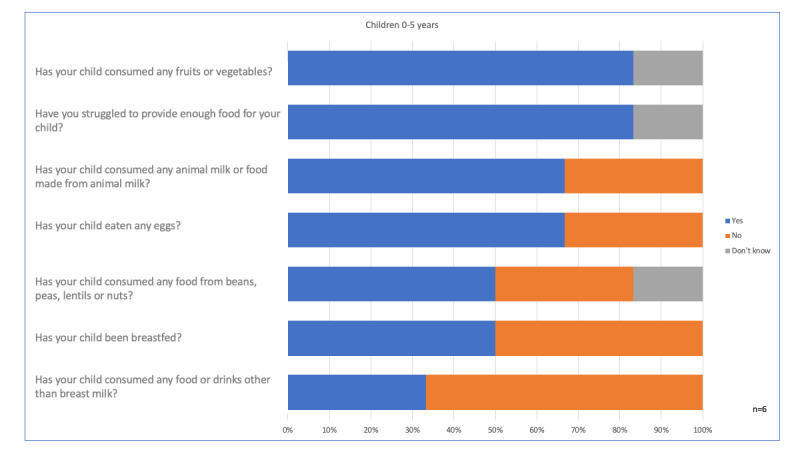
Nutrition aspects collected by mobile surveying.

**Figure 11:**
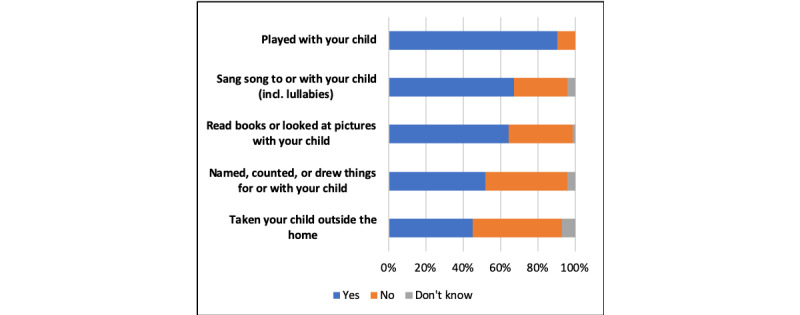
Early stimulation questions asked during the study period by mobile surveying.

Besides symptoms, we collected data on some dimensions of child development, including information on nutrition (Figure 10) and early stimulation (Figure 11). These data were collected for three villages. High-frequency nutrition data can help interpret symptoms and support recommendations to caregivers.

Figure 11 shows the data collected on children in terms of early stimulation. Early stimulation was shown to be crucial for the intellectual development of children. Although not directly related to emergency action, these data show how mobile surveys based on IVR can be used to explore a wide range of determinants of a child’s long-term well-being.

**Figure 12:**
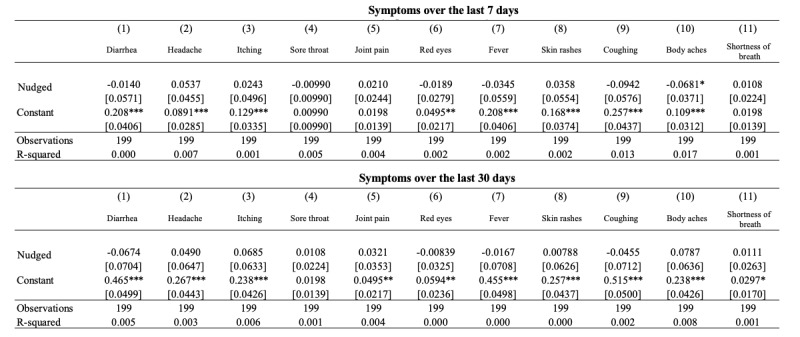
Linear regression for nudge participants and their symptoms. (***p<0.01, ** p<0.05, * p<0.1).

Finally, Figure 12 shows the results from the end-line investigation related to symptoms and nudges for the village participants.

## Discussion

### Principal Results

This study demonstrated the feasibility of the unprecedented use of a multimodal approach for collecting data related to child development in Malawi settings. The preliminary data collected through our different data streams showcase a feasible methodology for tracking children’s health and development in poor country settings at a high frequency.

### Comparison With Prior Work

While several studies applied in developing countries have already proven the feasibility of using wearable devices to understand how diseases behave and affect certain populations [38-41], including children [42], much less has been shown about the feasibility of multimodal approaches with the multiple data streams that we combined at the high frequency that we implemented within the complex setting that we targeted, which are all necessary elements to understand the intricacies of child development in poor countries and to unlock the potential of interventions powered by these data, with the potential to change the life trajectories of those children. The complexity and innovation of the study can be perceived by the scarcity of literature that describes the methods adopted with precision and reproducibility.

The execution of studies like this is still a challenge owing to the logistical and infrastructure issues that need to be overcome. However, the portability of the devices used favored the measurement of noninvasive biomarkers, such as EEG and ECG data, in environments without any structure, which is an important finding that was demonstrated. The EEG and ECG data showed that in addition to the feasibility of collection, the quality achieved has great potential for scaling interventions that previously did not consider these types of biomarkers. As seen in Figures 4 to 6, the quality of measurements indicated a feasible way to collect EEG readings using a wearable device, although further tests are still needed to find out whether the premise of the relationship of brain wave patterns with the involvement in infectious diseases or psychological trauma can be addressed by specific patterns. Toth et al [43] suggested the use of EEG combined with facial coding for improved evaluation of facial expressions. This type of approach could provide inputs for understanding stress levels through the assessment of facial patterns [44,45]. With regard to ECG data, studies [46-48] have demonstrated that this noninvasive biomarker can be used to measure malnutrition, in addition to the use of some specific standards for proxies related to temperature increase. The data from the proximity sensors indicated that nudges sent via SMS text messaging played a role in activating behavior change, and despite being an observation not yet reported in the literature, a similar approach used in Africa showed promising results for social network pattern studies [49]. Going further into this part, data from proximity sensors in combination with the syndromic data from mobile surveying could bring new insights into transmission routs for infectious diseases, supporting the understanding of how intrahousehold and interhousehold contacts play roles in disease spread. This sort of combination is important and opens up opportunities for future research involving extensive experiments on both modules. Synthetic models and simulations used to be reliable ways to create scenarios for understanding disease outbreaks, but periodically, reports from mobile surveys could really help in understanding which features of contact networks are the most important for disease spread. A doable app that can be mentioned involves the use of social networks for digital contact tracing of COVID-19 spreaders, measuring exposure and contagion, and complementing existing traditional disease surveillance systems.

### Limitations

The syndromic approach seems to be an important tool for assessing the risk and existence of outbreaks of infectious diseases in the studied region owing to the self-report of symptoms present in the children informed by their caregivers. The alignment of syndromic approaches with participatory surveillance plays an important role in understanding epidemiological settings at the community level [50], and even though there is a limitation in the implementation of more sophisticated tools, such as mobile apps, they are still strategic instruments for health surveillance systems. Nevertheless, the premise of the combination of not only information related to symptoms but also nutritional or developmental aspects has the possibility of deepening with the construction of syndemic models [51]. Even the observation of several factors that coexist in Malawi shows that they have been addressed as syndemics in similar contexts like Kenya or in similar social fabric related to food insecurity, maternal mental health, and domestic violence [52,53].

### Conclusions

The development of research that uses different technologies for data collection in children in the context of underdeveloped countries is challenging, requiring a recurrent search for instruments and strategies that enable and balance the needs and potential of certain areas [54]. Wearable technologies have demonstrated wide application in society, integrating the internet of things with telemetry systems to bring the context of remote management in the monitoring of health situations [55-57]. Even considering the observations mentioned by Piwek et al [58], where concerns related to security, reliability, and privacy in the use of these technologies are exposed, we conclude that the application of multimodal approaches like our approach is promising. In addition, they are able to achieve the immense challenge of collecting high-frequency data and feeding decision makers strategic information for planning policies and programs. Wearable technologies have already created a health revolution and are gradually reaching developed countries [59]. In addition, we understand that it is a good time for developing countries, even those that are in a critical scarcity scenario, to use this opportunity to leverage and accelerate the digitalization of health, bringing benefits to populations that lack new tools for understanding child well-being and development.

## Abbreviations

ECG: electrocardiography
EEG: electroencephalography
IRB: institutional review board
IVR: interactive voice response
